# Supplementary material for: Structure of human TRPV4 in complex with GTPase RhoA
Source: Nat Commun. 2023 Jun 23;14:3733. doi: 10.1038/s41467-023-39346-z (PMC10290124; doi:10.1038/s41467-023-39346-z)
Supplement: Supplementary file 1 — Supplementary Information [file 41467_2023_39346_MOESM1_ESM.pdf]

Supplementary Information

Structure of human TRPV4 in complex with GTPase RhoA

Kirill D. Nadezhdin[1,5], Irina A. Talyzina[1,2,5], Aravind Parthasarathy[3], Arthur Neuberger[1], David X. Zhang[3,4], Alexander I. Sobolevsky[1,*]

[1] Department of Biochemistry and Molecular Biophysics, Columbia University, New York, NY 10032, USA

[2] Integrated Program in Cellular, Molecular and Biomedical Studies, Columbia University, New York, NY 10032, USA

[3] Department of Medicine, Cardiovascular Center, Medical College of Wisconsin, Milwaukee, WI 53226, USA

[4] Department of Pharmacology and Toxicology, Medical College of Wisconsin, Milwaukee, WI 53226, USA

[5] These authors contributed equally.

*Corresponding author. E-mail: as4005@cumc.columbia.edu

This PDF file includes:

Supplementary Figures 1-10
Supplementary Table 1
Supplementary References

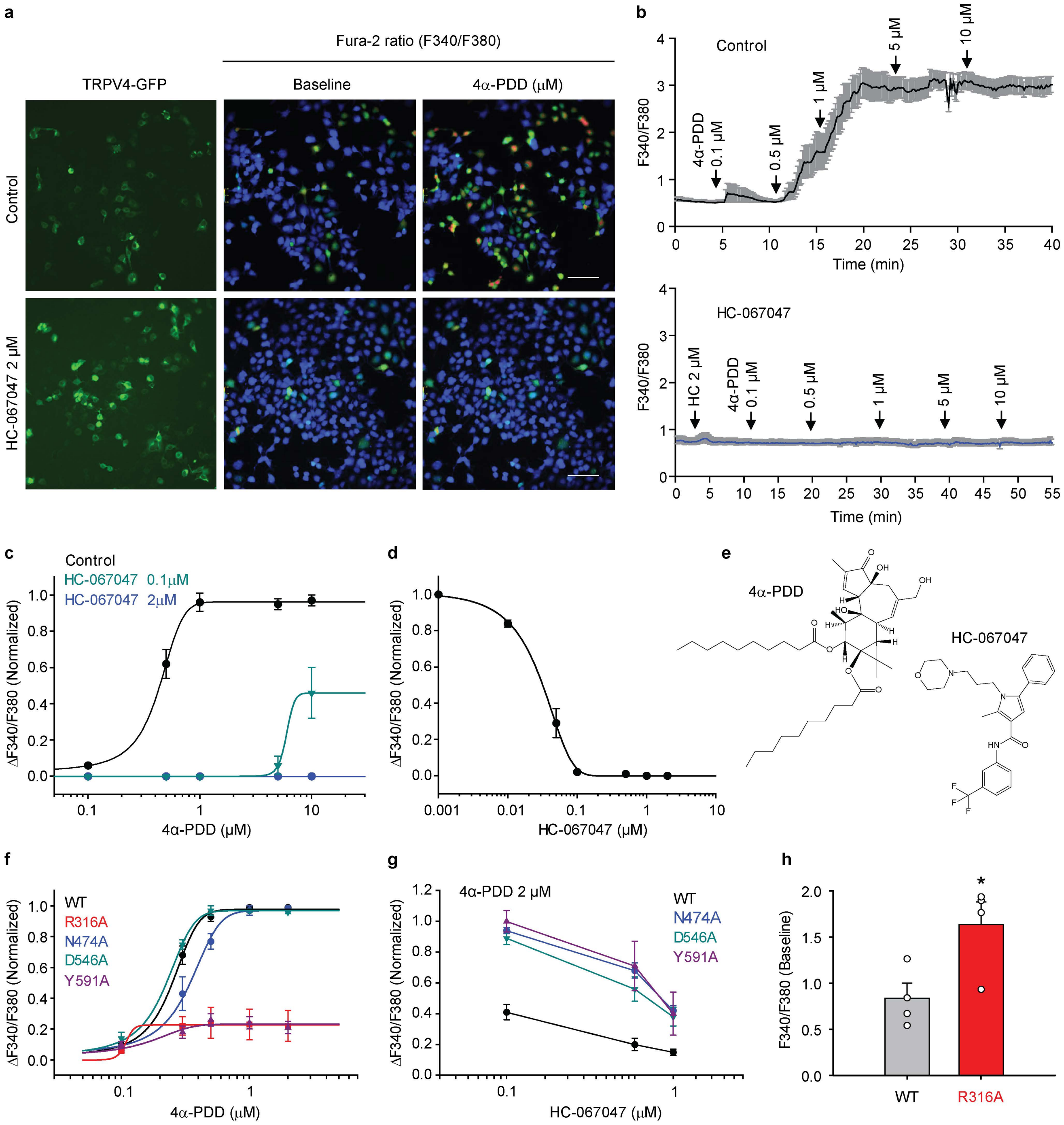

Supplementary Figure 1 | Functional characterization of wild type and mutant hTRPV4. a Representative GFP (left) and fura-2 (right 2 panels) fluorescence images of HEK293 cells transfected with wild-type TRPV4-GFP (C-terminal GFP fusion protein) before (baseline) and after addition of TRPV4 agonist 4α-PDD (10 μM), in the absence or presence of pretreatment with TRPV4 antagonist HC-067047 (2 μM), respectively. The intracellular Ca^{2+} concentrations were monitored by the ratiometric calcium indicator fura-2 and the results are presented as F340/F380 ratios, with the corresponding fluorescence pseudo-colored from blue to red on the scale of 0.0-5.0. Scale bar = 100 μm. The experiment was repeated four times. **b** Representative traces. The black or blue line denotes mean F340/F380 ratio of individual cells recorded in one assay and the gray area 1xSEM. 4α-PDD (0.1 μM – 10 μM) induced Ca^{2+} increase in a concentration-dependent manner, and this increase was largely inhibited by TRPV4-selective antagonist

HC-067047 (2 µM). **c** Dose-response curves for 4α-PDD in the absence or presence of HC-067047. The responses were normalized to the estimated maximal F340/F380 ratios in response to saturating concentrations of 4α-PDD. Curve fitting was performed using the ligand binding sigmoidal dose–response function (SigmaPlot version 12.5). The value of the half-maximal effective concentration for activation of hTRPV4 by 4α-PDD in the absence of HC-067047, EC_{50} = 450 ± 39 nM (n = 4), and > 2 µM (n = 4) after treatment with 2 µM HC-067047. **d** Dose-response curve for inhibition of hTRPV4 by HC-067047 in HEK293 cells stimulated with 4α-PDD (1 µM), with the half-maximal inhibitory concentration (IC_{50}) of 25 ± 6.2 nM (n = 4). **e** Chemical formulas for the TRPV4 agonist 4α-PDD and antagonist HC-067047. **f** Dose-response curves for 4α-PDD in wild-type and mutant TRPV4 channels (n = 4). **g** Dose-response curves for HC-067047 inhibition of 4α-PDD (2 µM)-induced activation of wild-type and mutant TRPV4 channels (n = 4). **h** Baseline F340/F380 ratios in wild-type and R316A mutant TRPV4 channels (n = 4). For comparison of baseline F340/F380 ratios, all cells expressing wild-type or mutant TRPV4-GFP channels were included in the analysis. Statistical analysis: two-sided t-test, * $P < 0.05$. Source data for panels **c**, **d**, **f**, **g**, and **h** are provided as a Source Data file. Data are presented as mean values ± SEM.

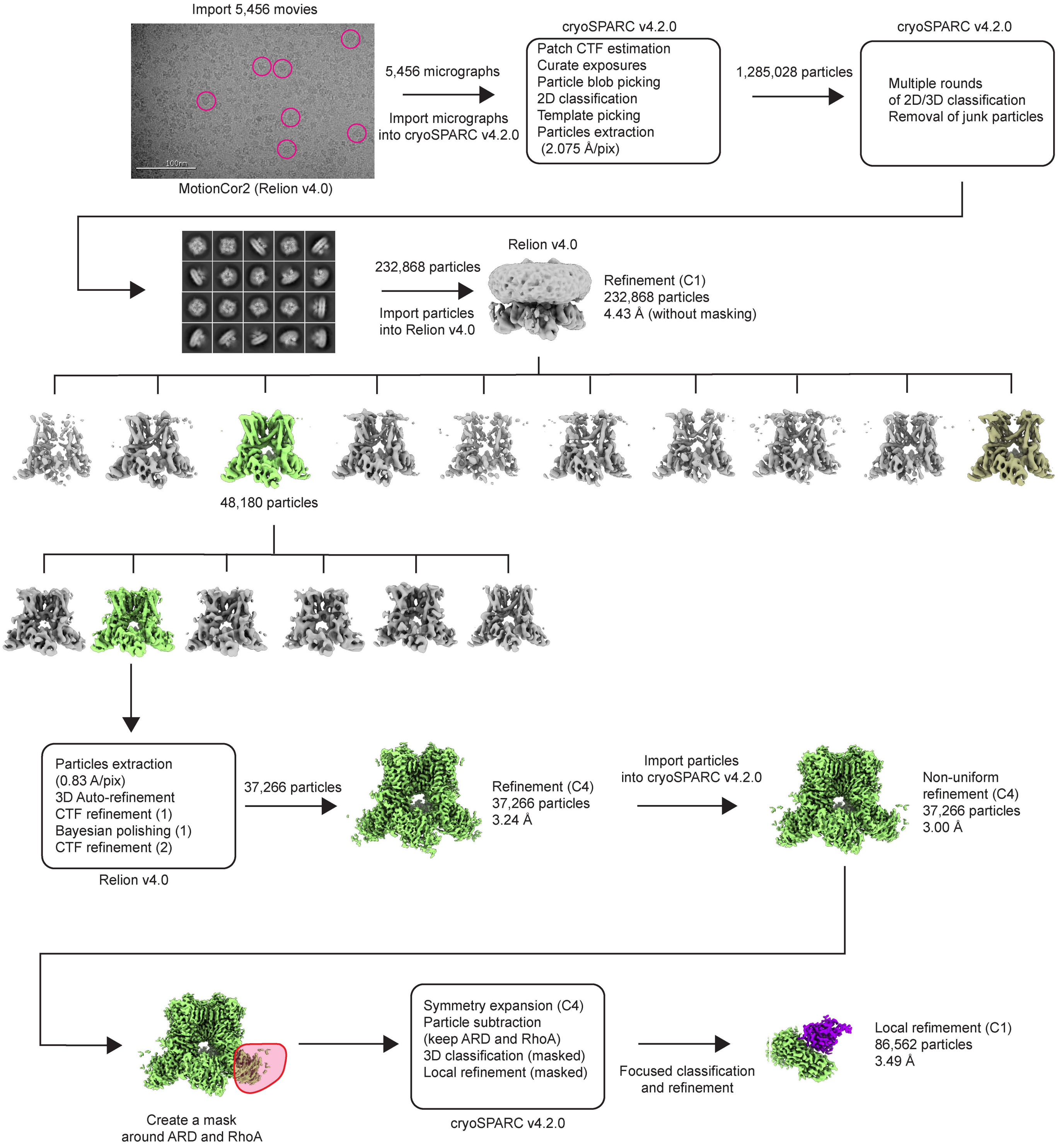

Supplementary Figure 2 | Overview of cryo-EM data collected for hTRPV4$_{apo}$ and 3D reconstruction workflow. In the representative micrograph, example particles are circled in pink.

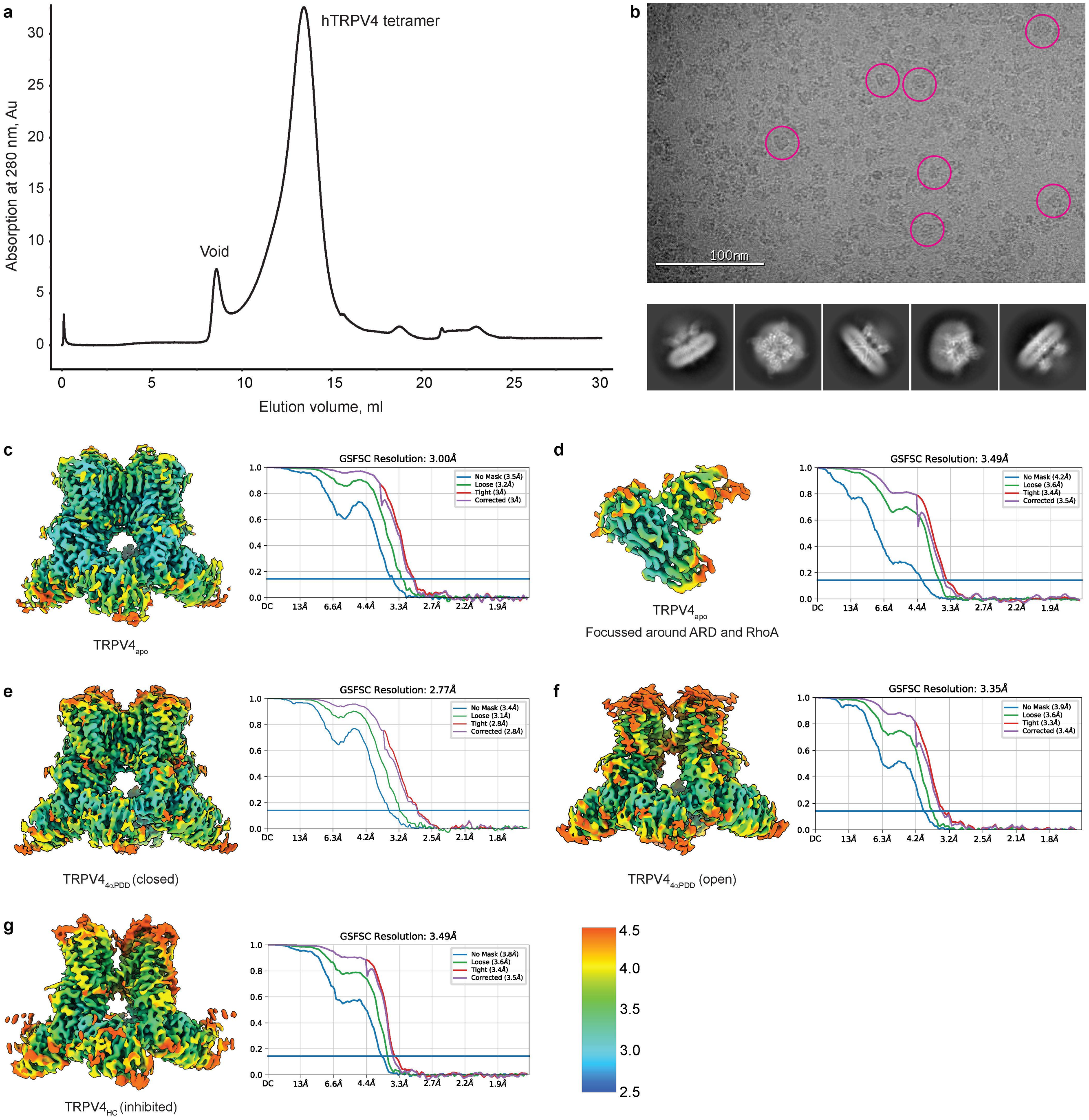

Supplementary Figure 3. Characteristics of Cryo-EM reconstructions | a SEC profile for TRPV4 in GDN. **b** Exemplar micrograph and reference-free 2D-classification averages from TRPV4$_{apo}$ structure collection with individual particles circled in red. A total number of 5,456, 15,624, and 11,551 of such micrographs were collected in the apo condition, in the presence of 4α-PDD and in the presence of HC-067047, respectively. **c-g** Cryo-EM maps are colored according to the local resolution estimation in cryoSPARC with FSC = 0.5 criterion. Plots show FSC curves calculated between half maps, with the overall resolution estimated using the FSC = 0.143 criterion.

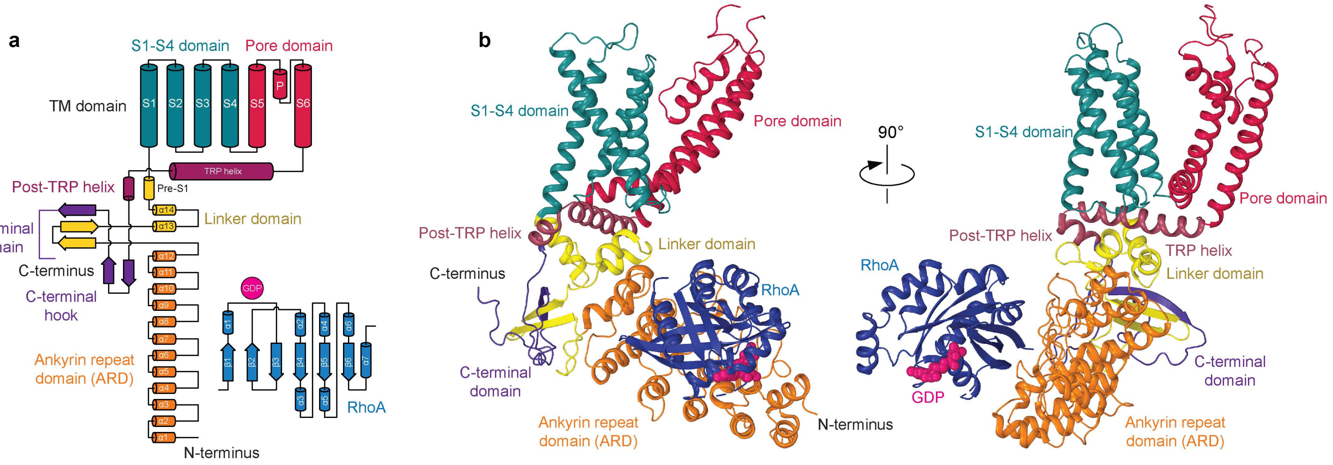

Supplementary Figure 4 | Overall architecture of the hTRPV4 subunit and RhoA. a hTRPV4 subunit topology diagram colored according to domain organization. **b** Cartoon representation of hTRPV4 subunit colored according to domain organization.

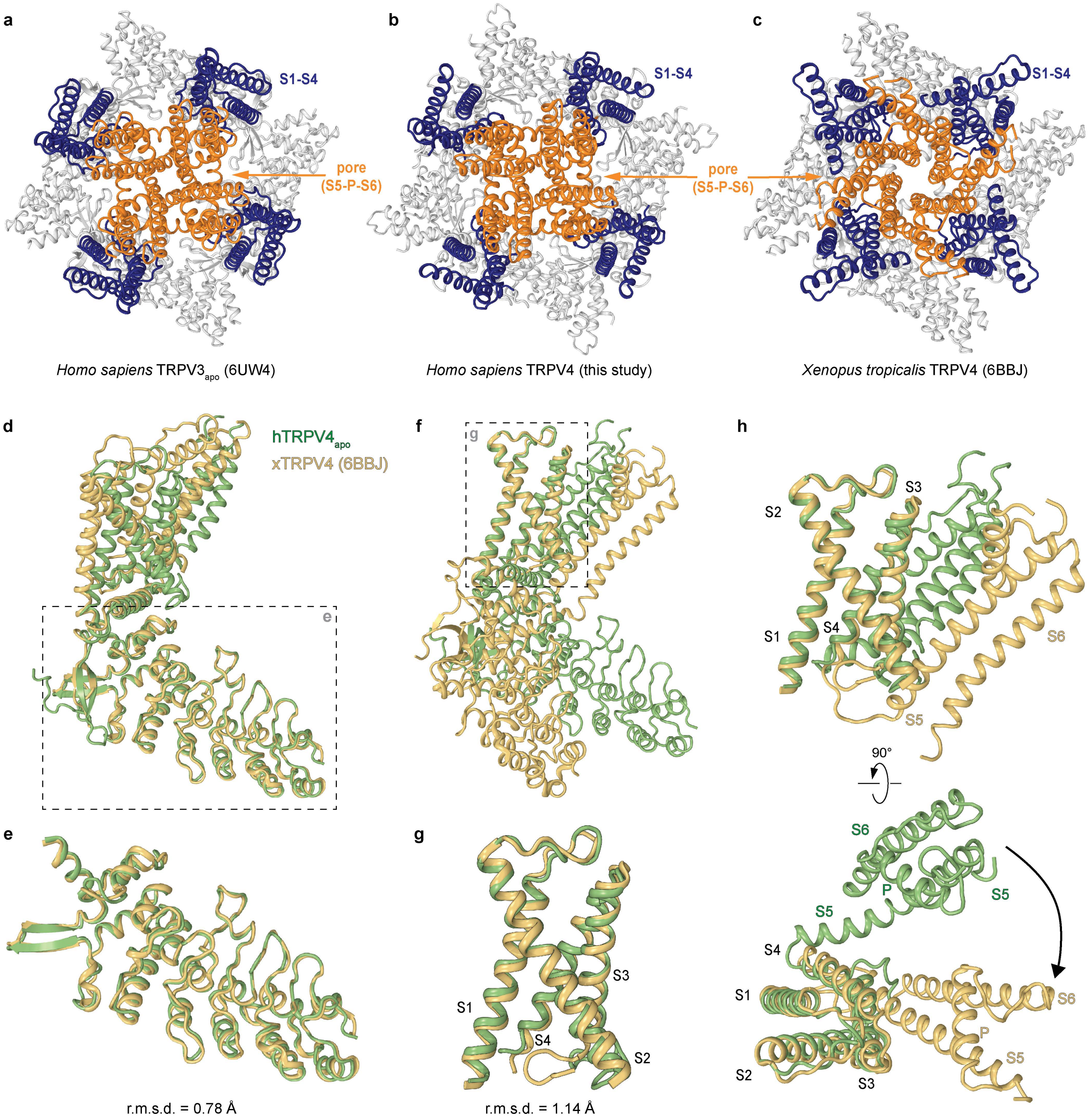

Supplementary Figure 5 | Structural comparisons of human TRPV4 with human TRPV3 and frog TRPV4. a-c Extracellular views of hTRPV3 (**a**, PDB ID: 6UW4)[1], hTRPV4$_{apo}$ (**b**, this study), and *Xenopus tropicalis* TRPV4 (**c**, PDB ID: 6BBJ)[2] aligned based on the ARDs. The colored grey, S1-S4 domains blue, and pore domains comprising S5-P-S6 – orange. **d** ARD-based superposition of single subunits from hTRPV4$_{apo}$ (green) and xTRPV4 (beige). **e** Close-up view of the ARDs from (**d**). **f** S1-S4 domain-based superposition of single subunits from hTRPV4$_{apo}$ and xTRPV4. **g** Close-up view of the S1-S4 domains from (**f**). **h** Close-up view of the entire TMDs from (**f**). Arrows show relative displacements of the pore domain relative to the S1-S4 domain. Note, while the overall architecture of hTRPV3, hTRPV4, and xTRPV4 (**a-c**) and individual domain structures (**e,g**) appear to be similar, there is a dramatic relative rearrangement of domains in hTRPV4 and xTRPV4 exemplified by the particularly striking displacement of the pore versus S1-S4 domains (**h**).

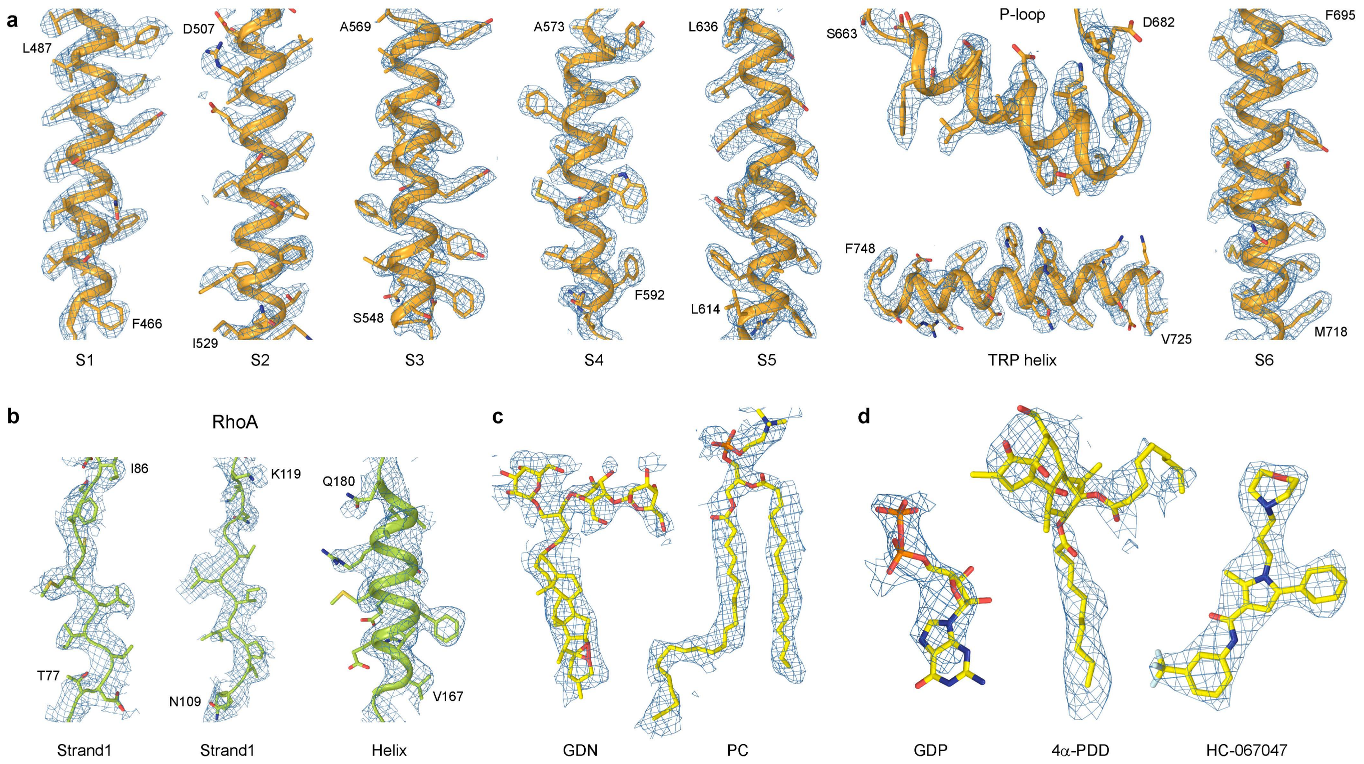

Supplementary Figure 6 | Cryo-EM density of hTRPV4 and RhoA. a Fragments of the $hTRPV4_{apo}$ TMD with the structural model shown as orange ribbon and sticks and the corresponding cryo-EM density as a blue mesh. **b** Fragments of the Rho[illegible] ctural model shown as green ribbon and sticks and the corresponding cryo-EM density as a blue mesh**. c** Representative densities (blue mesh) for the detergent glyco-diosgenin (GDN) and lipid phosphatidylcholine (PC) shown in sticks (yellow). **d** Representative densities (blue mesh) for the hTRPV4-RhoA ligands guanosine diphosphate (GDP), 4α-PDD, and HC-067047 shown in sticks.

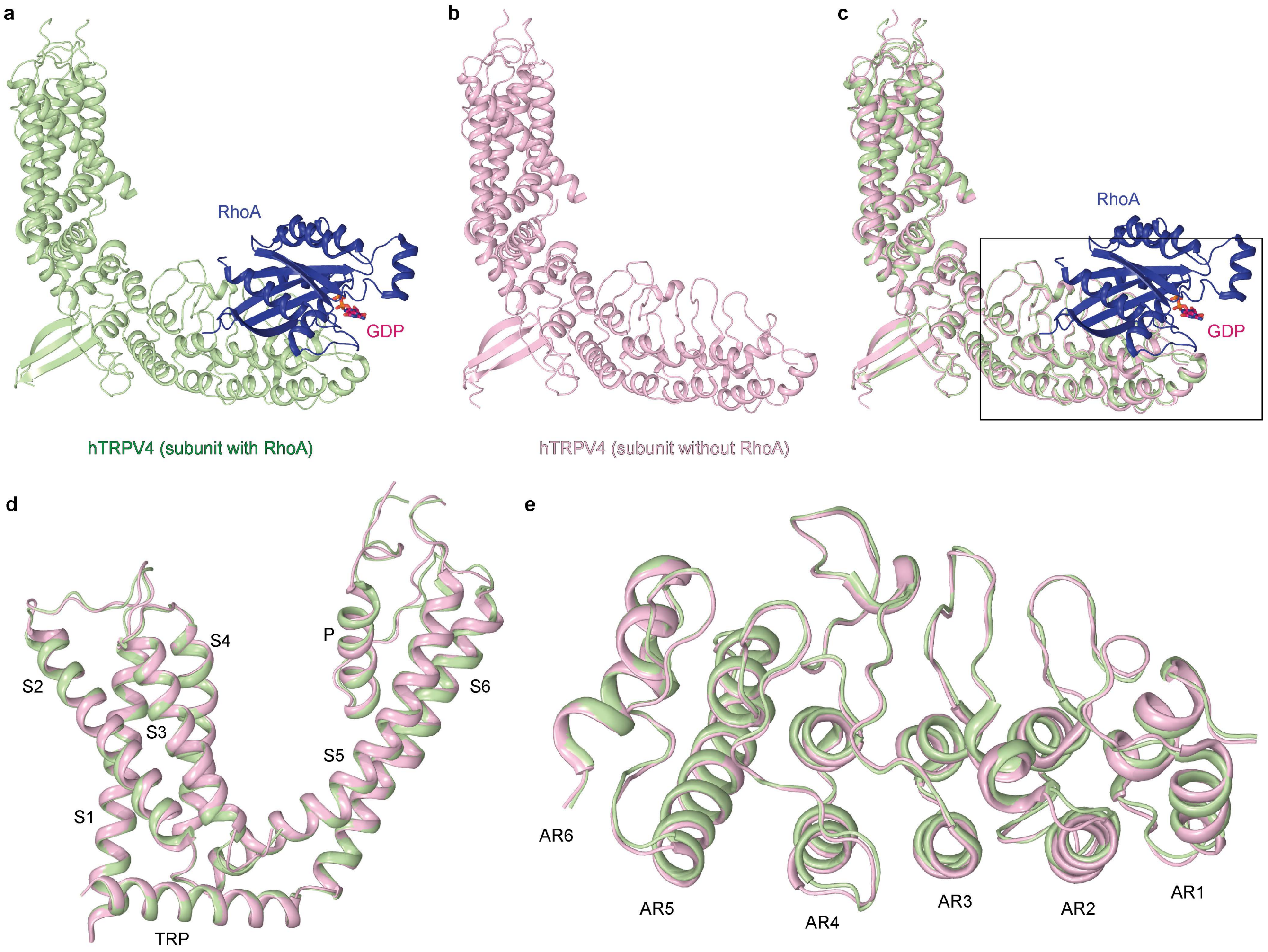

Supplementary Figure 7 | Comparison of hTRPV4 subunits with and without bound RhoA. a-c Single hTRPV4 subunit with (**a**) and without bound RhoA (**b**) and their superposition (**c**). **d-e** Close-up view of the TMD (**d**) and ARD (**e**) from superposition in (**c**).

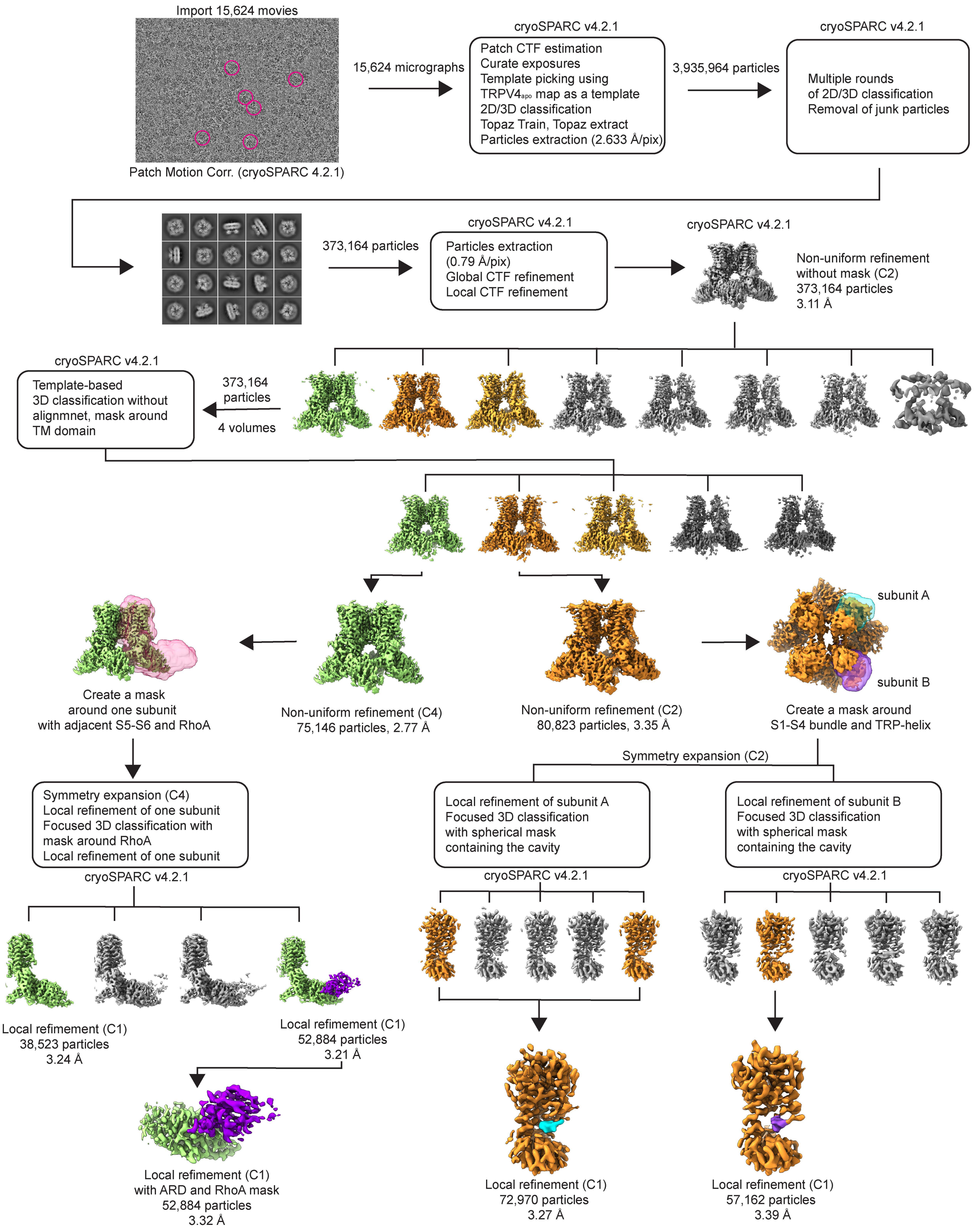

Supplementary Figure 8 | Overview of cryo-EM data collected for hTRPV4$_{4\alpha\text{-PDD}}$ and 3D reconstruction workflow. In the representative micrograph, example particles are circled in pink.

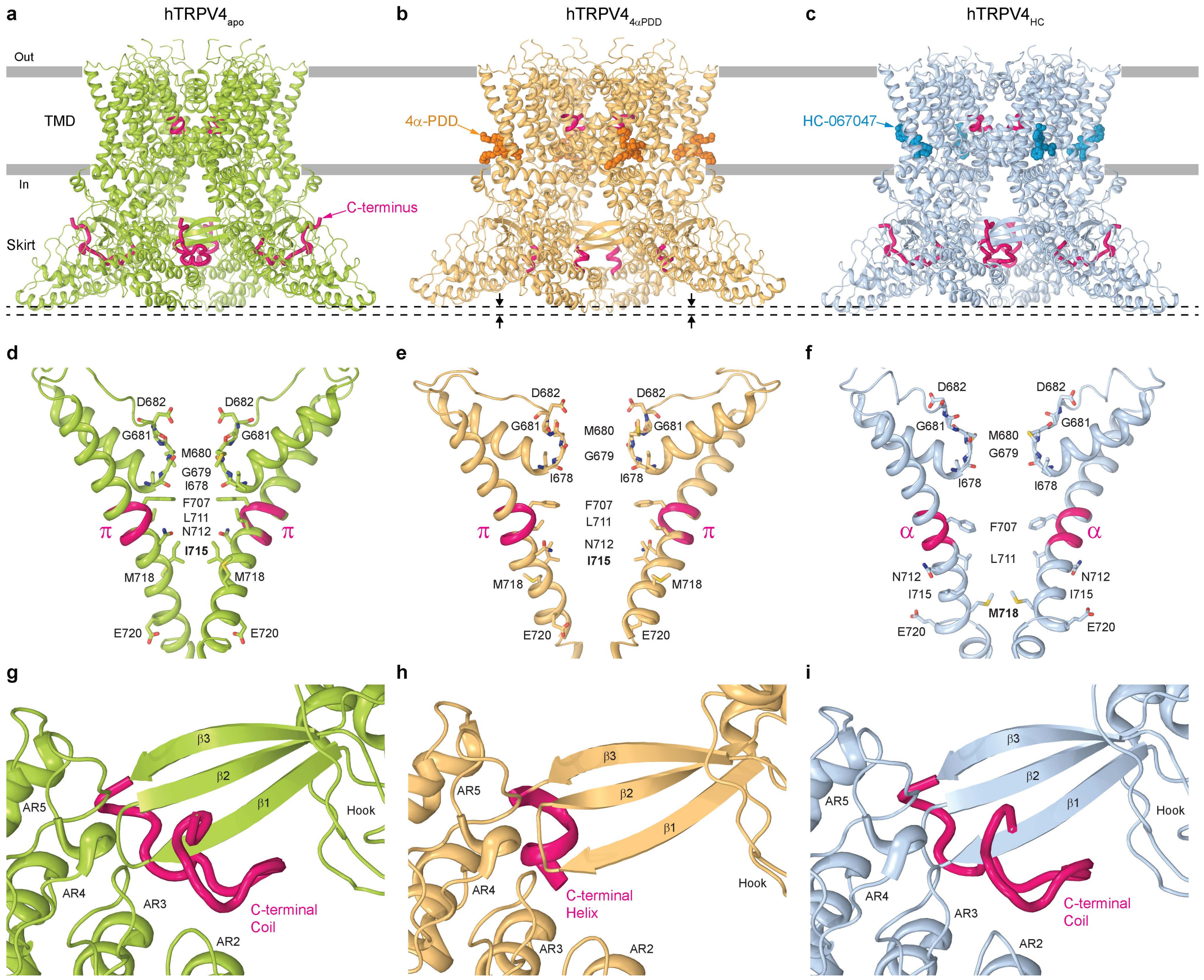

Supplementary Fig. 9 | Comparison of the hTRPV4$_{apo}$, hTRPV4$_{4αPDD}$ and hTRPV4$_{HC}$ structures. a-c hTRPV4$_{apo}$ (**a**), hTRPV4$_{4αPDD}$ (**b**) and hTRPV4$_{HC}$ (**c**) structures viewed parallel to the membrane, with the C-termini and regions of S6 undergoing the α-to-π transition colored pink. Molecules of 4α-PDD (**b**) and HC-067047 (**c**) are shown as space-filling models. Movement of the skirt towards the membrane in hTRPV4$_{4αPDD}$ (**b**) is indicated. **d-f** Pore-forming domains in hTRPV4$_{apo}$ (**d**), hTRPV4$_{4αPDD}$ (**e**) and hTRPV4$_{HC}$ (**f**) with the residues contributing to pore lining shown as sticks. Only two (A and C) of four subunits are shown, with the front and back subunits omitted for clarity. The region that undergoes the α-to-π transition in S6 (pink) is labeled. **g-i** Interface between the neighboring subunits in hTRPV4$_{apo}$ (**g**), hTRPV4$_{4αPDD}$ (**h**) and hTRPV4$_{HC}$ (**i**) with the C-terminus (pink) adapting coiled (**g**, **i**) or helical (**h**) conformations.

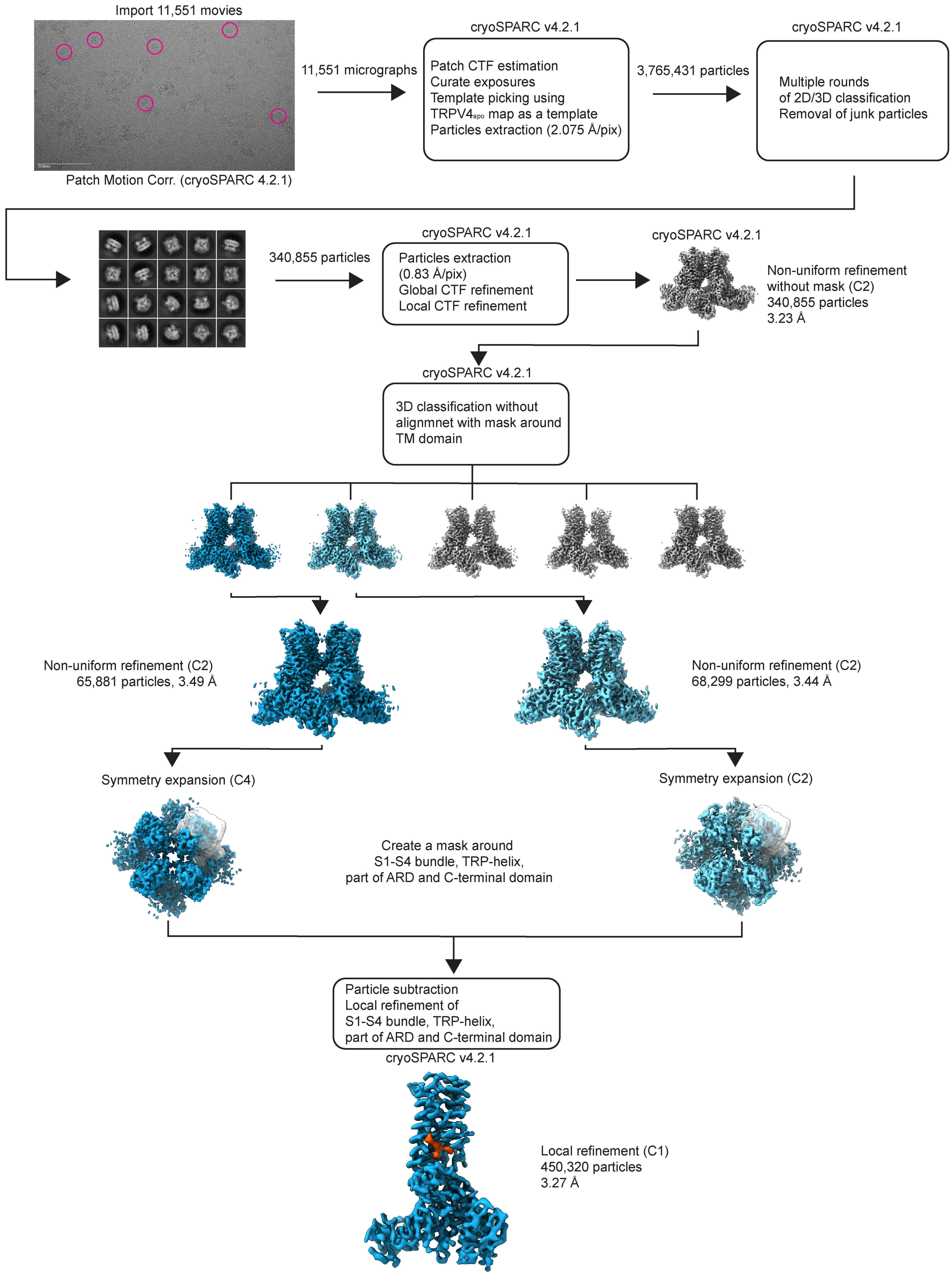

Supplementary Figure 10 | Overview of cryo-EM data collected for hTRPV4$_{HC}$ and 3D reconstruction workflow. In the representative micrograph, example particles are circled in pink.

Supplementary Table 1 | Cryo-EM data collection, refinement, and validation statistics.

**Structure**	**TRPV4$_{apo}$**	**ARD-RhoA**	**hTRPV4$_{4αPDD}$**	**hTRPV4$_{4αPDD}$**	**TRPV4$_{HC}$**
State	Closed		Open	Closed	Inhibited
Condition/Ligand	Apo	Focused	4α-PDD	4a-PDD	HC-067047
EMDB accession code	EMDB-40958	EMDB-40959	EMDB-40960	EMDB-40961	EMDB-40962
PDB accession code	8T1B	8T1C	8T1D	8T1E	8T1F
**Data collection and processing**					
Magnification	105,000x	105,000x	29,000x	29,000x	105,000x
Voltage (kV)	300	300	300	300	300
Electron exposure (e^{-}/Å2)	58	58	60	60	58
Defocus range (μm)	−0.5 to −1.5	−0.5 to −1.5	−0.75 to −1.5	−0.75 to −1.5	−0.5 to −1.5
Reported pixel size (Å)	0.83	0.83	0.7888	0.7888	0.83
Calibrated pixel size (Å)	0.83	0.83	0.7888	0.7888	0.83
Exposures (no.)	5,456	5,456	15,624	15,624	11,551
**Processing software**					
Platform software for particle picking	CryoSPARC 4.2.0	CryoSPARC 4.2.0	CryoSPARC 4.2.1	CryoSPARC 4.2.1	CryoSPARC 4.2.1
Motion correction	MotionCor2	MotionCor2	Patch Motion	CryoSPARC	CryoSPARC
CTF estimation	Patch CTF	Patch CTF	Patch CTF	Patch CTF	Patch CTF
Software for 2D/3D class. & refinements	Relion 4.0, CryoSPARC 4.2.0	Relion 4.0, CryoSPARC 4.2.0	CryoSPARC 4.2.1	CryoSPARC 4.2.1	CryoSPARC 4.2.1
Symmetry imposed	C4	C1	C2	C4	C2
Initial particle images (no.)	1,285,028	1,285,028	3,935,964	3,935,964	3,267,981
Final particle images (no.)	37,266	86,562	80,823	75,146	65,881
Map resolution (Å) FSC 0.143	3.00	3.49	3.35	2.77	3.49
Map resolution range, min/median/max (Å)	2.68/6.00/45.50	1.76/5.57/55.32	2.94/6.98/46.81	1.67/7.01/43.18	3.01/6.94/37.71
**Refinement**					
Initial models used (PDB code)	7AA5	1FTN	This study	This study	This study
Model resolution (Å) FSC threshold	3.00	3.49	3.35	2.77	3.49
Map sharpening *B* factor (Å^2)	−85.5	−127.5	−72.8	−78.6	−95.4
Model composition					
Non-hydrogen atoms	23,180	6040	20,338	22,083	20,790
Protein residues	2,700	377	2,502	2,516	2550
Ligand atoms	2,412	40	192	1871	344
R.m.s. deviations					
Bond lengths (Å)	0.006	0.006	0.006	0.007	0.006
Bond angles (°)	1.158	1.203	1.236	1.433	1.207
Validation					
CC (across whole map volume)	0.4893	0.3813	0.5624	0.4967	0.6085
CC (only across atoms in the model)	0.6731	0.6633	0.7449	0.7067	0.7786
Clashscore, all atoms	3.84	4.47	5.97	3.26	4.73
Poor rotamers (%)	0.00	0.31	0.45	0.18	0.71
Ramachandran plot					
Favored (%)	87.93	81.40	87.15	87.2	11.64
Allowed (%)	11.45	17.25	12.21	12.4	11.64
Disallowed (%)	0.62	1.31	0.64	0.32	0.63

Supplementary References

1 Deng, Z. *et al.* Gating of human TRPV3 in a lipid bilayer. *Nature Structural & Molecular Biology* **27**, 635-644, doi:10.1038/s41594-020-0428-2 (2020).

2 Deng, Z. *et al.* Cryo-EM and X-ray structures of TRPV4 reveal insight into ion permeation and gating mechanisms. *Nature Structural & Molecular Biology* **25**, 252-260, doi:10.1038/s41594-018-0037-5 (2018).
