# Supplementary material for: Structure of human TRPV4 in complex with GTPase RhoA
Source: Nat Commun. 2023 Jun 23;14:3733. doi: 10.1038/s41467-023-39346-z (PMC10290124; doi:10.1038/s41467-023-39346-z)
Supplement: Supplementary file 3 — Description of Additional Supplementary Files [file 41467_2023_39346_MOESM3_ESM.pdf]

File name: Supplementary Movie 1
Description: Conformational changes during hTRPV4 activation. Shown is a morph between the closed apo-state $hTRPV4_{apo}$ and open-state $hTRPV4_{4\alpha PDD}$ that illustrates conformational changes in hTRPV4 accompanying channel opening in response to agonist 4α-PDD binding.

File name: Supplementary Movie 2
Description: Conformational changes during hTRPV4 inhibition. Shown is a morph between the closed apo-state $hTRPV4_{apo}$ and inhibited state $hTRPV4_{HC}$ that illustrates conformational changes in hTRPV4 accompanying inhibition by the antagonist HC-067047.
